# Comparison of Treatment Outcomes Between First-Line Chemotherapy With or Without Bevacizumab for Advanced Ovarian Clear Cell Carcinoma (Tohoku Gynecologic Cancer Unit: TGCU-RS001A Study)

**DOI:** 10.3390/cancers16223801

**Published:** 2024-11-12

**Authors:** Tadahiro Shoji, Eriko Takatori, Takayuki Nagasawa, Masahiro Kagabu, Tsukasa Baba, Tatsuhiko Shigeto, Yukiko Matsumura, Dai Shimizu, Yukihiro Terada, Manabu Seino, Tsuyoshi Ohta, Satoru Nagase, Shogo Shigeta, Hideki Tokunaga, Muneaki Shimada, Michiko Kaiho-Sakuma, Shigenori Furukawa, Shu Soeda, Takafumi Watanabe, Fumiaki Takahashi, Yoshihito Yokoyama

**Affiliations:** 1 Department of Obstetrics and Gynecology, Iwate Medical University School of Medicine, Yahaba 028-3695, Japan; takatori@iwate-med.ac.jp (E.T.); tnagasaw@iwate-med.ac.jp (T.N.); mkagabu@iwate-med.ac.jp (M.K.); babatsu@iwate-med.ac.jp (T.B.); 2 Department of Obstetrics and Gynecology, Hirosaki University School of Medicine, Hirosaki 036-8563, Japan; t-shigeto@hirosaki-u.ac.jp (T.S.); y-matsu@hirosaki-u.ac.jp (Y.M.); yokoyama@hirosaki-u.ac.jp (Y.Y.); 3 Department of Obstetrics and Gynecology, Akita University Graduate School of Medicine, Akita 010-8543, Japan; shimizud@doc.med.akita-u.ac.jp (D.S.); teraday@doc.med.akita-u.ac.jp (Y.T.); 4 Department of Obstetrics and Gynecology, Yamagata University Faculty of Medicine, Yamagata 990-9585, Japan; m-seino@med.id.yamagata-u.ac.jp (M.S.); oota-t@med.id.yamagata-u.ac.jp (T.O.); nagases@med.id.yamagata-u.ac.jp (S.N.); 5 Department of Obstetrics and Gynecology, Tohoku University Graduate School of Medicine, Sendai 980-8574, Japan; shogo.shigeta.a4@tohoku.ac.jp (S.S.); hideki.tokunaga.a1@tohoku.ac.jp (H.T.); muneaki.shimada.b7@tohoku.ac.jp (M.S.); 6 Department of Gynecology, Miyagi Cancer Center, Natori 981-1293, Japan; michiko-kaihou@miyagi-pho.jp; 7 Department of Obstetrics and Gynecology, Fukushima Medical University School of Medicine, Fukushima 960-1295, Japan; s-furu@infoseek.jp (S.F.); s-soeda@fmu.ac.jp (S.S.); t-wata@fmu.ac.jp (T.W.); 8 Department of Information Science, Iwate Medical University, Yahaba 028-3694, Japan; ftakahas@iwate-med.ac.jp

**Keywords:** ovarian cancer, clear cell carcinoma, first-line chemotherapy, maintenance therapy, bevacizumab

## Abstract

**Objective**: The usefulness of bevacizumab (BEV) as first-line chemotherapy for advanced ovarian clear cell carcinoma (CCC) was retrospectively evaluated at Tohoku Gynecologic Cancer Unit institutions. **Patients and Methods**: A total of 81 patients (52 patients without BEV and 29 with BEV) with advanced ovarian CCC who received initial platinum-based chemotherapy were enrolled. We selected 26 patients each without and with BEV according to propensity score matching methods, and compared the platinum-resistant recurrence rate, response rate, progression-free survival (PFS), overall survival (OS), and adverse events between the two groups. **Results**: The objective response rates for the patients with measurable disease in the non-BEV and BEV groups were 60.0% and 95.5%, respectively (*p* = 0.007). The recurrence of platinum-resistant patients was observed in 50.0% of the non-BEV group and 23.1% of the BEV group (*p* = 0.117). The median PFS was 12 and 22 months, respectively (*p* = 0.034), and the median OS was 32 and 47 months, respectively (*p* = 0.223). In the BEV group, grade 3 hypertension and proteinuria occurred in two patients, and thrombosis and carboplatin hypersensitivity occurred in one patient. Grade 4 gastrointestinal perforation occurred in one patient. **Conclusions**: First-line chemotherapy with BEV in advanced CCC showed the potential for high response rates and improved PFS.

## Simple Summary

We evaluated the efficacy of bevacizumab (BEV) in first-line chemotherapy for clear cell carcinoma (CCC). We categorized patients with advanced clear cell carcinoma (CCC) who received platinum-based first-line chemotherapy into a non-BEV group and a BEV group, and compared the progression-free survival (PFS), overall survival (OS), response rates, adverse events, and platinum agent-resistant recurrence rates. The PFS was improved and response rates were higher in the BEV group. One patient in the BEV group had grade 4 gastrointestinal perforation, although no treatment-related death was reported. First-line chemotherapy with BEV in advanced CCC showed the potential for high response rates and an improved PFS.

## 1. Introduction

The incidence and number of patients with ovarian cancer has increased annually, and the current incidence is approximately 1.3 times higher than that reported for patients ten years ago [1]. Ovarian cancer is the third most common gynecologic malignancy, following cervical and endometrial cancer. In 2020, approximately 314,000 new cases of ovarian cancer and 207,000 deaths were reported worldwide [2]. In contrast, in the United States, it is estimated that in 2020, 19,710 new cases of ovarian cancer were diagnosed, and 13,270 deaths. Ovarian cancer deaths exceed those from endometrial and cervical cancer [3].

Clear cell carcinoma (CCC) is a rare subtype of epithelial ovarian cancer in the United States, although it accounts for 24.8% of all epithelial ovarian cancers in Japan [4,5,6,7]. The majority of CCC cases are diagnosed at an early stage, and advanced cases are rare. The Japanese Gynecologic Oncology Group (JGOG) 3017 trial reported that only 156 of 667 (23.3%) patients diagnosed with CCC were in the advanced stage [8].

Generally, CCC has a poorer prognosis than high-grade serous carcinoma, particularly in advanced-stage patients with lower sensitivity to platinum-based chemotherapy [6,8,9,10,11,12]. CPT-11 + cisplatin (CDDP) therapy failed to improve the progression-free survival (PFS) of patients receiving paclitaxel + carboplatin (TC) therapy in the JGOG3017 trial. Therefore, this regimen is used as a first-line chemotherapy option in clinical practice [8]. In addition, the GOG268 trial was a phase II international clinical trial of TC in combination with temsirolimus as maintenance therapy for stage III/IV ovarian CCC. A total of 90 patients were recruited to the study: 45 in the US and Korea and 45 in Japan. The median PFS was 11 months for the US and Korea and 12 months for Japan. This regimen did not statistically significantly increase the PFS at 12 months compared to historical controls [13]. Thus, the TC regimen is the most potent cytotoxic regimen reported in the literature thus far [8]. Accordingly, there is an urgent need for novel therapeutic strategies for advanced CCC.

In the GOG218 and ICON7 trials, the incorporation of bevacizumab (BEV) into platinum-based regimens, followed by maintenance therapy, resulted in an extension of the PFS by 3.8 months and 1.7 months, respectively [14,15]. However, in ICON7, where over 50% of the CCC patients were diagnosed with early-stage ovarian cancer, the efficacy of BEV in individuals with advanced CCC could not be definitively established [16].

In Japan, BEV was approved to treat advanced ovarian cancer in November 2013, leading to an increase in its use. A single-center retrospective study reported that the addition of BEV to initial chemotherapy for advanced CCC extended the PFS compared with the group without BEV [17].

In the JGOG3022 trial, a prospective observational cohort study in Japanese patients, combination therapy with TC and BEV for CCC showed a 63.6% (n = 11) response rate, suggesting the possibility of significant benefit from BEV [18]. Furthermore, our previous study showed that the inclusion of BEV in primary chemotherapy for advanced ovarian cancer prolonged the PFS and overall survival (OS), and also reduced platinum-resistant recurrence rates [19]. To our knowledge, this was the first report to highlight the benefit of BEV in advanced ovarian cancer in a Japanese population. In contrast, there are very limited reports which compare BEV administered versus no BEV for advanced CCC.

The present study retrospectively evaluated the effectiveness of BEV in the treatment of advanced CCC at Tohoku Gynecologic Cancer Unit (TGCU) institutions.

## 2. Subject and Methods

### 2.1. Patients and Treatment Groups

Eighty-one patients diagnosed with stage III/IV CCC according to the International Federation of Gynecology and Obstetrics [FIGO 2014] classification [20] and who received initial platinum-based chemotherapy at seven TGCU institutions between November 2013 and March 2024 were included in this study. Patients were divided into two groups: the BEV group (n = 29) and the non-BEV group (n = 52). The primary endpoint was PFS, and secondary endpoints were anti-tumor response, OS, adverse events (AEs), and platinum-resistant recurrence rate.

Patients in the BEV group were defined with the following treatment criteria: (1) those who received platinum-based chemotherapy with BEV after primary debulking surgery, followed by BEV maintenance therapy (BEV-throughout treatment); or (2) those who received platinum-based chemotherapy with BEV after interval debulking surgery (IDS), followed by BEV maintenance therapy. Groups (1) and (2) were defined as those who received at least three cycles of platinum-based chemotherapy with BEV.

Patients with clear findings of carcinomatous peritonitis on diagnostic imaging, adenocarcinoma cells detected on ascitic fluid cytology, and serum CA125 levels of 70 U/mL or higher who underwent neoadjuvant chemotherapy (NACT) were also included. Patients who had received poly ADP-ribose polymerase (PARP) inhibitors as maintenance therapy for initial and recurrence treatment were excluded.

### 2.2. Treatment

This study used a primary chemotherapy regimen of a combination of taxanes and platinum-based agents. Paclitaxel (175 mg/m^2^) plus carboplatin (area under the curve [AUC]5–6) or docetaxel (70 mg/m^2^) plus carboplatin (AUC5–6) was administered every 3 weeks. These chemotherapies were administered for six to nine cycles. However, switching to cisplatin was permitted if the patient developed carboplatin hypersensitivity. In that case, triweekly paclitaxel (175 mg/m^2^) and cisplatin (50 mg/m^2^) were administered. Bevacizumab was administered at 15 mg/kg every three weeks for 21 cycles. Treatment was discontinued when disease progression or AEs occurred that precluded the continuation of treatment.

### 2.3. Efficacy and Safety

Anti-tumor efficacy was evaluated according to RECIST ver. 1.1 [21]. The occurrence and severity of AEs and treatment-related AEs were evaluated according to the Common Toxicity Criteria for Adverse Events ver. 5.0 JCOG Japanese version (CTCAE ver5.0-JCOG) [22]. The follow-up plan after completion of the first treatment was according to the Japanese ovarian cancer treatment guidelines. Imaging examinations were performed regularly, and additional imaging examinations were performed as needed when CA125 was abnormal. Recurrence was diagnosed using computed tomography (CT) or positron emission tomography (PET).

### 2.4. Statistical Analysis

To account for the nonrandomized treatment administration of bevacizumab, we used a propensity score matching method to reduce the effects of confounding. The individual propensity score for receipt of bevacizumab treatment was estimated with the use of a multivariable logistic regression model that included age, stage, PDS and IDS. Matching was performed with the use of a 1:1 matching protocol without replacement (greedy matching algorithm), with a caliper width equal to 0.2 of the standard deviation of the logit of the propensity score.

The data cut-off date was 31 March 2024. Statistical significance was calculated for patient background, anti-tumor efficacy, and AEs using the chi-square test. Progression-free survival and OS were calculated from the start of the first treatment to the documented date of progression, death, or last follow-up, whichever occurred first. The impact of treatment on survival was assessed by constructing Kaplan–Meier curves using a log-rank test. The hazard ratio and associated 95% confidence interval (CI) were calculated with the use of a stratified Cox proportional hazards model. In addition, multivariate analysis using a stratified Cox proportional hazards model was performed to demonstrate the consistency of the treatment effect in the prespecified subgroups.

All statistical analyses were performed using EZR ver. 1.68 (Saitama Medical Center, Jichi Medical University, Saitama, Japan), a graphical user interface for R 2.9-1 (R Foundation for Statistical Computing, Vienna, Austria). More precisely, it is a modified version of R commander designed to incorporate statistical functions commonly used in biostatistics [23].

## 3. Results

### 3.1. Patient Characteristics

Of the 81 patients in the total population (52 with BEV and 29 without BEV), 52 patients (26 with BEV and 26 without BEV) were selected by propensity score matching methods. The patient characteristics of the patients before propensity score matching is shown in Supplementary Table S1 and after matching in Table 1. The clinical stage of the propensity score-matched patients were adjusted to equal numbers of stage III and IV.

In the group without BEV, 16 patients were complete and optimal while 9 were suboptimal during the first surgery. In contrast, 14 patients were complete and optimal, whereas 11 were suboptimal in the BEV group.

Both groups included one patient each who received NACT. Interval debulking surgery was performed in five patients in the group without BEV, with two and three patients showing complete, optimal surgery. In the BEV group, seven patients underwent IDS, and surgical completion was complete, optimal, and suboptimal in one, five, and one patient, respectively.

### 3.2. Treatment and Anti-Tumor Response

Supplementary Table S2 shows the treatment and anti-tumor response before propensity score matching and Table 2 shows it after matching. In the group without BEV, all 26 patients received TC therapy. All 26 patients in the BEV group received TC + BEV therapy. However, one patient switched to paclitaxel + cisplatin (TP) + BEV therapy due to grade 3 carboplatin hypersensitivity.

The median number of platinum-based chemotherapy cycles was 6 (Range: 2–9) in the group without BEV and 6 in the BEV group (Range: 5–15). The median number of BEV cycles was 8.5 (Range: 2–22).

At the start of initial chemotherapy, the number of patients with measurable disease was 15 in the group without BEV and 22 in the group with BEV. The anti-tumor responses in the non-BEV group were complete response (CR) in six cases, partial response (PR) in three cases, and progressive disease (PD) in six cases. In contrast, 10 patients in the BEV group had CR, 11 had PR, and 1 had PD. The objective response rates for the patients with measurable disease were 60.0% and 95.5%, respectively (*p* = 0.007) (Table 2).

### 3.3. Adverse Events

Table 3 shows a comparison of the adverse events in each group after propensity score matching methods.

Non-hematological toxicity events of grade 3 or higher in the group without BEV were grade 3 cerebral infarction in one patient (3.8%) and grade 4 gastrointestinal perforation in one patient (3.8%). On the other hand, in the BEV group, peripheral neuropathy was observed in three patients (11.5%), grade 3 hypertension and urinary protein in two patients (7.7%), grade 3 thrombosis in one patient (3.8%), and grade 4 gastrointestinal perforation (GIP) in one patient (3.8%). Furthermore, one patient (3.8%) in the BEV group had grade 3 carboplatin hypersensitivity. The patient developed carboplatin hypersensitivity during the eighth cycle of TC + BEV therapy and was subsequently switched to TP + BEV therapy. There were no patients with treatment-related deaths.

### 3.4. Recurrence Pattern

The recurrence pattern before propensity score matching is shown in Supplementary Table S3 and after matching is shown in Table 4. A proportion of 20 of the 26 patients in the group without BEV (76.9%) and 15 of the 26 patients in the BEV group (57.7%) experienced recurrence. Of these, 7 (26.9%) and 13 (50.0%) patients in the group without BEV exhibited platinum sensitivity and resistance recurrence, respectively, while nine (34.6%) and six (23.1%) patients were in the BEV group for these recurrence patterns (*p* = 0.117). The median platinum-free interval (PFI) for 20 patients in the group without BEV and 15 patients in the BEV group with recurrent disease was 4.5 months (Range: 0–29) and 7 months (Range: 0–40), respectively (*p* = 0.270) (Table 4).

### 3.5. Survival Analysis

The median follow-up period for the 52 patients selected by propensity score matching methods was 29 months (Range: 2–106).

**Figure 1:**
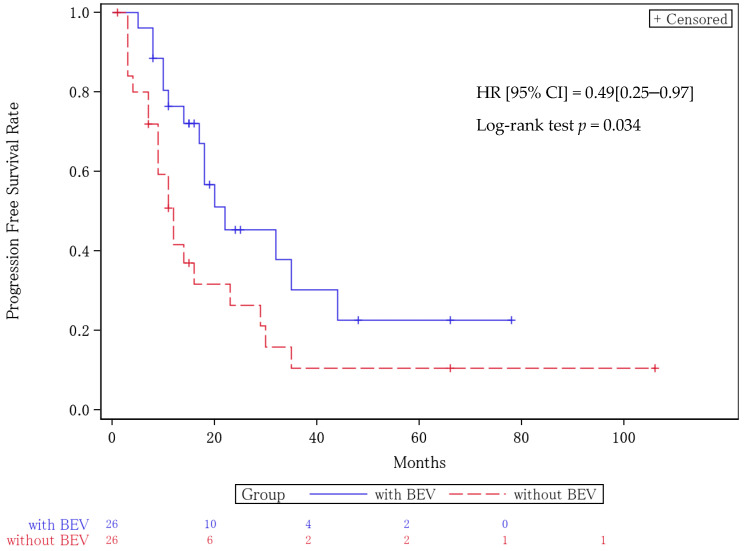
Kaplan–Meier curves for progression-free survival. The median PFS for patients who did not receive BEV was 12 months (95% CI, 9–23), while the median PFS for patients who received BEV was 22 months (95% CI, 17–44). The hazard ratio was 0.49 (95% CI = 0.25–0.97), with a *p* value of 0.034.

**Figure 2:**
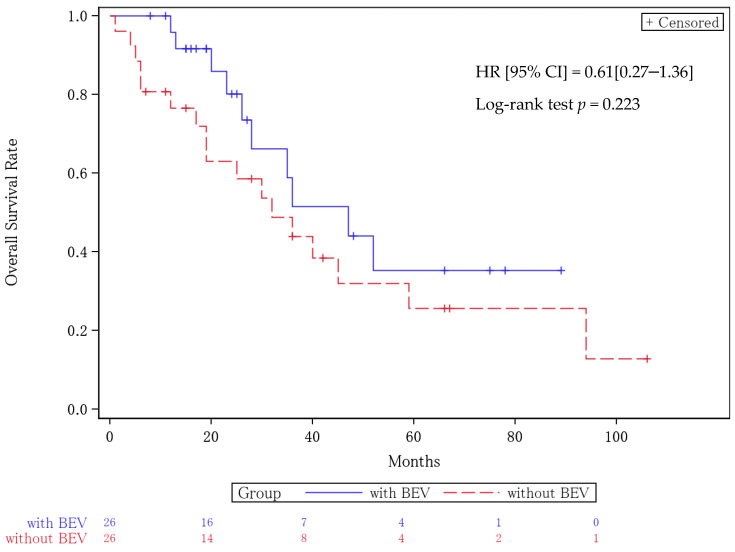
Kaplan–Meier curves for overall survival. Median OS for patients who did not receive BEV was 32 months (95% CI, 17–59), while the median PFS for patients who received BEV 47 months (95% CI, 26; not reached). The hazard ratio was 0.61 (95% CI = 0.27–1.36) with a *p* value of 0.223.

The median PFS for the patients who did not receive BEV was 12 months (95% CI, 9–23), while the median PFS for the patients who received BEV was 22 months (95% CI, 17–44). The hazard ratio (HR) was 0.49 (95% CI = 0.25–0.97), with a *p* value of 0.034 (Figure 1). The median OS was 32 months (95% CI, 17–59) in the BEV non-treated group and 47 months (95% CI, 26- not reached) in the BEV group. The HR was 0.61 (95% CI = 0.27–1.36) with a *p* value of 0.223 (Figure 2). BEV administration was extracted as a prognostic factor in each of the multivariate analyses for the PFS and OS (Table 5).

## 4. Discussion

There are limited reports comparing the recurrence rates, PFS, and OS in advanced CCC with or without BEV as first-line chemotherapy. This paucity of data is due to the rarity of CCC in Western countries and Europe and the fact that it is almost always diagnosed at an early stage [4,16].

A retrospective study examining the impact of BEV on CCC showed that the inclusion of BEV in salvage chemotherapy regimens for recurrence CCC was associated with increased response rates and an improved PFS [24]. Similarly, BEV monotherapy has been associated with a higher clinical benefit rate (45%) and prolonged PFS in recurrent ovarian CCC [25]; however, there is no suggestion of an improved OS. The present retrospective study was designed to investigate the benefits of BEV as first-line chemotherapy for advanced ovarian CCC.

Tate et al. compared the PFS and OS in patients with advanced ovarian CCC treated with first-line chemotherapy, including 18 patients treated with BEV and 10 patients without BEV. The progression-free survival with and without BEV was 29.8 and 12.0 months, respectively, indicating an additive effect of BEV (*p* = 0.036). However, the median OS was 49.6 and 30.0 months, respectively, failing to demonstrate a benefit of BEV (*p* = 0.464) [17]. However, the patients in this study were compared before 2013 for the non-BEV group and after 2013 for the BEV group, which remained a problem. Our study also showed a prolonged PFS but not OS in the BEV group compared to the non-BEV group. Our study compared patients after 2013 in both groups, which was considered to be a more reliable method.

The analysis of data from the JGOG3107 trial showed that the rates of platinum-resistant recurrence in the TC and CPT-11 + CDDP therapy groups were 40.3% and 48.3%, respectively [26]. In our study, the platinum-resistance recurrence rate in the group without BEV was 50.0%. These differences may be because the JGOG3017 trial included stages I–IV, whereas our study included only stages III/IV. In contrast, the rate of platinum-resistant recurrence in the BEV group was very low (23.1%), suggesting that BEV was beneficial.

If a patient is diagnosed with platinum-resistant recurrence, the options for subsequent chemotherapy are limited, and the OS is not expected to be prolonged. If the platinum-resistant recurrence rate was reduced, we expected the OS in the BEV group to be prolonged due to there being more options for second-line chemotherapy. However, due to the reduced number of patients using propensity score matching methods, it was not possible to demonstrate an OS benefit in the BEV group. The response rates were 95.5% and 60.0% for the groups with and without BEV, respectively (*p* = 0.007). The median PFI for the patients with recurrence was 7 months in the BEV group and 4.5 months in the BEV-naïve group (*p* = 0.270). These results may demonstrate the prolongation of the OS if the number of patients is increased. One patient was switched to TP + BEV therapy after the eighth cycle due to grade 3 carboplatin hypersensitivity. This patient underwent IDS and had a PFS of 22 months and OS of 36 months. Both were within the 95% CI, and we considered this to not affect our evaluation of this study.

Aggressive surgery may be justified as an initial treatment owing to the low sensitivity of CCC to chemotherapy. Aggressive surgery for advanced CCC is effective for histopathological cancers other than CCC [27]. In this study, the PFS was 10 months longer in the BEV group than in the non-BEV group, despite similar completion rates of PDS and IDS in each group. This result was strongly suggestive of the benefits of adding BEV to the chemotherapy regimen.

In the GOG218 and ICON7 trails, no exacerbations of hematologic toxicity due to BEV were reported [14,15]. Therefore, this study focused only on non-hematologic toxicity. In the present study, grade 3 or higher hypertension and urinary protein in the BEV group were observed in 7.6% of the patients, respectively. On the other hand, the reported rates in the JGOG3022 trial were 23.2% and 12.6%, respectively [18]. This is because our study was a retrospective study based on medical records and may not have picked up AEs. In the BEV group, there was one case each of grade 3 or 4 thrombosis and GIP, although they were manageable. Grade 3 peripheral neuropathy was observed only in the BEV group. BEV has been reported to be associated with the potential to exacerbate peripheral neuropathy induced by paclitaxel in breast cancer patients receiving chemotherapy [28], and in another report, 26% of patients treated with the combination of paclitaxel and BEV had grade 3 or higher peripheral neuropathy [29]. Although our study specifically investigated platinum-based chemotherapy, similar findings were observed in ovarian cancer patients treated with taxanes.

The authors reviewed and reported clinical trials using novel agents for CCC [30]. TAS-117 is an AKT inhibitor and is currently in phase I clinical trials for CCC. MDM2 is involved in the degradation of TP53 and expressed more frequently in CCC than other histopathologic types. MDM2 inhibitors have demonstrated anti-tumor effects in vitro and in vivo in CCC cell lines without TP53 mutations, and clinical trials using MDM2 inhibitors are ongoing [31]. In addition, the JGOG is planning a prospective observational study (JGOG 3033 trial) on TC plus BEV as first-line chemotherapy for advanced CCC. Furthermore, it will be interesting to determine how the use of PARP inhibitors as an initial maintenance therapy for CCC will change the platinum-resistant recurrence rate and further prolong the PFS and OS. We look forward to the establishment of evidence for ovarian cancer treatment in Japan.

This study had several limitations. Firstly, because this study was retrospective, the evidence level is not considered strong. Secondly, due to concerns about emergency management in the event of gastrointestinal perforation, two of the seven TGCU institutions did not administer BEV to any patients. Thirdly, there were no standardized BEV dosing criteria at TGCU, and the administration of BEV was determined based on the criteria of each institution. Additionally, surgical procedures were not standardized at each institution, which may have affected the results. Fourthly, treatment after recurrence was not standardized and differed at each institution. Lastly, the number of cases required to detect a hazard ratio of 0.545 (MST: 22 vs. 12 months) with 24 months of enrollment, 48 months of follow-up, a one-sided significance level of 5%, and 80% power is 38 cases per group and 67 expected events, which was slightly under-powered. In addition, the actual detection power with 26 cases was about 66 percent.

## 5. Conclusions

First-line chemotherapy with BEV for advanced CCC showed the potential to improve the PFS with a high response rate without severe toxicity. To our knowledge, these results are the first reported in the world for a Japanese population with the same study period. We expect Japanese multicenter clinical trials to demonstrate an improvement in the OS. Additionally, identifying biomarkers for better therapeutic outcomes would be interesting for future research.

## Figures and Tables

**Table 1:** Patient characteristics (propensity score-matched patients).

		Without BEV (N = 26)	With BEV (N = 26)	*p* Value
Age	Median, Range	58 (37–74)	54.5 (37–77)	0.827 **
Diagnosis	Ovarian	26	26	1.000 *
	Peritoneal	0	0	
Stage	III	25 (96.2%)	25 (96.2%)	1.000 *
	IV	1 (3.9%)	1 (3.9%)	
PDS	Complete	7 (26.9%)	6 (23.1%)	0.878 *
Completion	Optimal	9 (34.6%)	8 (30.8%)	
	Suboptimal	9 (34.6%)	11 (42.4%)	
NACT		1 (3.8%)	1 (3.8%)	1.000 *
IDS	Yes	5 (19.2%)	7 (26.9%)	0.510 *
	No	21 (80.8%)	19 (73.1%)	
IDS	Complete	2 (40.0%)	1 (14.3%)	0.462 *
Completion	Optimal	3 (60.0%)	5 (71.4%)	
	Suboptimal	0	1 (14.3%)	

PDS, primary debulking surgery; IDS, interval debulking surgery; NACT, neoadjuvant chemotherapy; BEV, bevacizumab; * Chi-squared test; ** Wilcoxon rank sum test.

**Table 2:** Treatment and anti-tumor response (propensity score-matched patients).

		Without BEV (N = 26)	With BEV (N = 26)	*p* Value
No. of platinum cycles		6 (2–9)	6 (5–15)	0.006 **
No. of BEV cycles		0	8.5 (2–22)	
No. of patients with measurable lesions		15	22	0.032 *
Tumor response	CR	6 (40.0%)	10 (45.5%)	
	PR	3 (20.0%)	11 (50.0%)	
	SD	0 (0.0%)	0 (0.0%)	
	PD	6 (40.0%)	1 (4.5%)	
	ORR	9 (60.0%)	21 (95.5%)	0.007 *

BEV, bevacizumab; CR, complete response; PR, partial response; SD, stable disease; PD, progressive disease; ORR, objective response rate; * Chi-squared test; ** Wilcoxon rank sum test.

**Table 3:** Adverse events.

	Without BEV (N = 26)				With BEV (N = 26)				*p* Value (≥Grade 3)
	G1	G2	G3	G4	G1	G2	G3	G4	
Peripheral neuropathy	10	1	0	0	8	5	3	0	0.235 *
Hypertension	2	0	0	0	4	5	2	0	0.490 *
Proteinuria	1	1	0	0	2	2	2	0	0.490 *
Thromboembolic event	0	0	1	0	0	0	1	0	1.000 *
GIP	0	0	0	1	1	0	0	1	1.000 *
CBDCA hypersensitivity	0	0	0	0	0	0	1	0	1.000 *

GIP, gastrointestinal perforation; CBDCA, carboplatin; BEV, bevacizumab; G, grade; * Chi-squared test.

**Table 4:** Recurrence pattern (propensity score-matched patients).

	Without BEV (N = 26)	With BEV (N = 26)	*p* Value
Patients with recurrence	20 (76.9%)	15 (57.7%)	0.139 *
Median PFI (Range)	4.5 (0–29)	7 (0–40)	0.270 **
Platinum-resistant	13 (50.0%)	6 (23.1%)	0.117 *
Platinum-sensitive	7 (26.9%)	9 (34.6%)	

BEV, bevacizumab; PFI, platinum-free interval; * Chi-squared test; ** Wilcoxon rank sum test.

**Table 5:** Multivariate analysis of treatment-related factors.

A. Progression-Free Survival				
**Factor**		***p* Value**	**HR**	**95% CI**
Bevacizumab	With/without	0.0001	0.284	0.149–0.540
Age		0.0097	0.969	0.946–0.992
PDS	Complete, optimal/suboptimal	<0.0001	0.181	0.090–0.366
IDS	Complete, optimal/suboptimal	0.0003	0.237	0.108–0.521
**B. Overall Survival**				
**Factor**		***p* Value**	**HR**	**95% CI**
Bevacizumab	With/without	0.0012	0.264	0.118–0.591
Age		0.0331	0.971	0.945–0.998
PDS	Complete, optimal/suboptimal	<0.0001	0.110	0.048–0.250
IDS	Complete, optimal/suboptimal	0.0016	0.251	0.106–0.593

PDS, primary debulking surgery; IDS, interval debulking surgery; HR, hazard ratio; CI, confidence interval.

## Data Availability

The data presented in this study are available in this article and Supplementary Materials.

## Supplementary Materials

The following supporting information can be downloaded at: https://www.mdpi.com/article/10.3390/cancers16223801/s1, Table S1: Patient characteristics (Unmatched patients); Table S2: Treatment and Anti-Tumor Response (Unmatched patients); Table S3: Recurrence pattern (Unmatched patients).
