# Supplementary material for: Comparison of Treatment Outcomes Between First-Line Chemotherapy With or Without Bevacizumab for Advanced Ovarian Clear Cell Carcinoma (Tohoku Gynecologic Cancer Unit: TGCU-RS001A Study)
Source: Cancers (Basel). 2024 Nov 12;16(22):3801. doi: 10.3390/cancers16223801 (PMC11593229; doi:10.3390/cancers16223801)
Supplement: Supplementary file 1 [file cancers-16-03801-s001.zip › cancers-3283190-supplementary.pdf]

Supplementary Table S1. Patient characteristics (Unmatched patients).

		Without BEV (N=52)	With BEV (N=29)	*p* value
Age	Median, Range	56 (27-75)	58 (37-77)	0.171**
Diagnosis	Ovarian	51	29	1.000*
	Peritoneal	1	0	
Stage	III	38 (73.1%)	28 (96.6%)	0.008*
	IV	14 (26.9%)	1 (3.4%)	
PDS Completion	Complete	10 (19.2%)	7 (24.1%)	0.781*
	Optimal	13 (25.0%)	8 (27.6%)	
	Suboptimal	28 (53.8%)	13 (44.8%)	
NACT		1 (1.9%)	1 (3.4%)	1.000*
IDS	Yes	8 (15.4%)	9 (31.0%)	0.153*
	No	44(84.6%)	20 (69.0%)	
IDS Completion	Complete	4(50.0%)	2(22.2%)	0.637*
	Optimal	3(37.5%)	6(66.7%)	
	Suboptimal	1(12.5%)	1(11.1%)	

PDS primary debulking surgery, IDS interval debulking surgery, NACT neoadjuvant chemotherapy, BEV bevacizumab,

* Chi-squared test, **Wilcoxon rank sum test

Supplementary Table S2. Treatment and Anti-Tumor Response (Unmatched patients).

		Without BEV (N=52)	With BEV (N=29)	*p* value
No of platinum cycles		6 (2-9)	6 (3-15)	0.002**
No of BEV cycles		0	9 (2-22)	
No of patients with measurable lesions		39	24	0.421*
Tumor response	CR	9 (23.1%)	11 (45.8%)	
	PR	6 (15.4%)	12 (50.0%)	
	SD	1 (2.6%)	0 (0.0%)	
	PD	23 (69.0%)	1 (1.6%)	
	ORR	15 (39.5%)	23 (95.8%)	< 0.001*

BEV bevacizumab, CR complete response, PR partial response, SD stable disease, PD progressive disease,

ORR objective response rate, *Chi-squared test, **Wilcoxon rank sum test.

Supplementary Table S3. Recurrence pattern (Unmatched patients).

	Unmatched Patients		
	Without BEV (N=52)	With BEV (N=29)	*p* value
Patients with recurrence	45（86.5%）	17（58.6%）	0.002*
Median PFI（Range)	1 (0-29)	9 (0-40)	0.003**
Platinum-resistant	34（65.4%）	6（ 20.7%）	< 0.001*
Platinum-sensitive	11（21.2%）	10（ 34.5%）	

BEV bevacizumab, PFI platinum-free interval, * Chi-squared test, **Wilcoxon rank sum test.
